# Socioeconomic position and employment status in patients with lung cancer – a register-based study

**DOI:** 10.2340/1651-226X.2025.44017

**Published:** 2025-09-18

**Authors:** Emma Brink, Marc Sampedro Pilegaard, Claus Vinther Nielsen, Pernille Pedersen

**Affiliations:** a Department of Public Health, Aarhus University, Aarhus, Denmark; b DEFACTUM, Central Denmark Region, Aarhus, Denmark; c Department of Social Medicine and Rehabilitation, Goedstrup Hospital, Goedstrup, Denmark

**Keywords:** Employment, rehabilitation, vocational, socioeconomic factors, lung neoplasms, cohort studies

## Abstract

**Background and purpose:**

Lung cancer patients have an increased risk of adverse employment outcomes. However, limited research exists on the association between socioeconomic position (SEP) and employment status in this cancer group. This study explored the influence of SEP on employment status after a lung cancer diagnosis.

**Patient/material and methods:**

This population-based cohort study included all working-age Danish residents diagnosed with lung cancer between 2000 and 2015. Logistic regression analyses were conducted to assess the association between socioeconomic variables (education, income, sick leave, and work status before diagnosis), and working and disability pension 3 years after diagnosis.

**Results:**

A total of 1,946 lung cancer patients were included. High income and long education were associated with higher odds of working, odds ratio (OR) = 2.31 (1.65–3.24) and OR = 1.92 (1.15–3.21), respectively, and lower odds of disability pension, OR = 0.19 (0.11–0.33) and OR = 0.30 (0.13–0.70), respectively. Moreover, sick leave and being out of work before diagnosis were associated with lower odds of working, OR = 0.25 (0.13–0.46) and OR = 0.32 (0.24–0.43), respectively, and higher odds of disability pension, OR = 3.73 (2.14–6.50) and OR = 2.88 (2.14–3.87), respectively.

**Interpretation:**

Lung cancer patients with low SEP are less likely to be employed and more likely to receive disability benefits. Therefore, rehabilitation to support socioeconomically disadvantaged lung cancer patients is needed.

## Introduction

Lung cancer is the second most common cancer globally and has been the primary cause of cancer-related deaths for many decades [1]. However, survival rates are increasing in many countries due to advanced treatments and earlier diagnosis [2, 3]. Unfortunately, the consequences of a cancer diagnosis reach beyond aspects of survival, as many patients with cancer experience unemployment, long-term sick leave, and disability pension in the years following the diagnosis [4–6]. Mainly, patients with lung cancer are at higher risk of adverse employment outcomes compared with other cancer types [7, 8].

A Danish register-based cohort study recently found that patients with lung cancer had the lowest chance of working among all cancer types at 1, 3, and 5 years after diagnosis [9]. Moreover, this study found that there could be a possible association between socioeconomic position (SEP) prior to cancer diagnoses and employment outcomes after cancer. The concept of SEP can vary significantly. It is typically assessed using one or a combination of indicators, each describing different and often interconnected aspects of an individual’s place in society [10]. The most commonly used indicators are related to education, income, occupation or employment, housing, and marital status or cohabitation [10–14].

Previous research has demonstrated that the incidence and mortality of lung cancer [13, 15] and the type of treatment patients with lung cancer receive [16] are related to the individual’s SEP. A Danish cohort study even showed that recent improvements in cancer survival rates primarily benefit individuals with high SEP, leading to an increasing gap between social groups [11]. Regarding the correlation between SEP and employment after cancer, studies on patients with breast and colorectal cancer have demonstrated that high income and long education were positively associated with rates of return to work [17, 18]. Moreover, unemployment and longer periods of sick leave before diagnosis were found to be negatively associated with employment after cancer in a cohort of mixed cancer patients [7]. However, research on the association between SEP and employment status among patients with lung cancer is limited to a few studies that have only assessed the impact of income [19–21]. Moreover, these findings have been inconsistent due to limitations such as small study populations and a high risk of selection bias [19–21]. Large-scale nationwide studies are needed to further assess the association between SEP prior to diagnosis and employment status after diagnosis in lung cancer patients. Such knowledge can be used to identify high-risk patients and provide further evidence of inequality in the cancer trajectory of patients with lung cancer.

The present study explored the association between SEP prior to diagnosis and employment status in patients with lung cancer 3 years after diagnosis.

## Patients/material and methods

### Study design and data sources

The present study is a population-based cohort study using data from Danish registers: The Danish Cancer Register (CAR) [22], Statistics Denmark (STD) [23], The Danish National Patient Register (DNPR) [24], and The Danish Register for Evaluation of Marginalization (DREAM) [25]. CAR has recorded all incident cancers in Denmark since 1943 and is characterized by a high degree of completeness and diagnostic validity due to mandatory reporting and systematic data collection [22]. DNPR contains data on all somatic inpatient visits since 1977 and on somatic outpatient and psychiatric visits since 1995 and has been validated for a range of diagnoses and procedures, with high positive predictive values reported [24]. For all Danish residents, the receipt of social benefits, such as unemployment benefits, sick leave benefits, and disability pension, is registered in DREAM. Each week, the type of social benefit is recorded if a person has received the benefit for at least 1 day. DREAM has been validated against workplace-registered data on sick leave and self-reported income source [25, 26], with both studies indicating that the register provides valid data. Every Danish resident is assigned a unique personal identity number, which is utilized to link data across all registers.

### Study population

All Danish residents diagnosed with a first-time diagnosis of lung cancer, between January 1^st^ 2000 and December 31^st^ 2015, were included. The study population was identified from an existing cohort from a previous study investigating the risk of receiving disability pension in all Danish cancer patients [27]. Eligibility in this study was based on the WHO International Classification of Diseases, 10^th^ revision (ICD-10) and defined as having received any of the following codes in CAR: C340-C349 [22, 28]. We included lung cancer patients between 20 and 60 years of age at the time of diagnosis, as the public retirement age was 65 years in Denmark during the study period. The baseline was defined as the date of diagnosis. We excluded all patients who had died or received disability pension before this date, and cases where the date of diagnosis was identical to the date of death, along with those who had other cancer diagnoses before lung cancer or were diagnosed with multiple cancer diagnoses on the same date, and those who had missing information on income and education.

### Exposures

We used four indicators to measure SEP: household income, educational level, sick leave 12–24 months before diagnosis, and work status 2 years before diagnosis. Information on income and education was retrieved from STD [23], and information on sick leave and work status was retrieved from DREAM [25]. Data on household income were used to divide the study population into three subgroups: low (<20,131€), medium (20,132€ – 40,263€), and high income (>40,264€), following the categorization used in a prior study [27]. The subgroups for educational level comprised the following five groups: primary/high school, vocational education, short further education, bachelor’s degree, and long further education. Sick leave was measured in weeks 12–24 months before diagnosis. A washout period of 12 months before diagnosis was selected to avoid the impact of sick leave associated with the cancer diagnosis. The amount of sick leave was then categorized into four subgroups: 0, 1–7, 8–27, and >28 weeks, reflecting short-, medium-, and long-term sick leave [26]. Finally, information on received social benefits 2 years before diagnosis was used to divide the study population into two subgroups based on their work status: working and not working.

### Outcomes

Employment status was investigated using two different outcomes: (1) Working (yes/no) and (2) Disability pension (yes/no). Both outcomes were assessed using the information provided by DREAM and estimated 3 years after diagnosis. Working was defined as being able to participate in the labor market, whether full- or part-time. Patients receiving unemployment benefits, flexible job compensation, or no social benefits were defined as working. Patients with lung cancer had to be in one of the categories above for 4 consecutive weeks until the week of measurement to be categorized as working. Patients receiving any of the remaining types of social benefits, including sick leave and disability pension, were categorized as not working. For the second outcome, any patient registered in DREAM as receiving disability pension during the week of measurement was categorized as such. The outcomes were only assessed in the lung cancer patients who were still alive and residing in Denmark after the 3 years of follow-up. All persons who died or emigrated were excluded from the analyses.

### Covariates

Age, gender, and comorbidities were included as covariates based on their known association with employment outcomes following cancer. This selection was informed by previous research demonstrating that higher age, female gender, and the presence of comorbidities are associated with a lower likelihood of returning to work after cancer [18, 29, 30]. These variables were included to improve precision of estimates and to account for differences in baseline prognosis [31]. Information on age and gender was retrieved from STD. Comorbidity status was assessed according to the Charlson comorbidity index [32] and based on information on 19 somatic comorbidities obtained from DNPR. The status was then dichotomized into having and not having comorbidities.

### Statistics

Descriptive statistics were used to describe the cohort based on the socioeconomic variables and covariates at baseline. The baseline characteristics are presented for three populations: the total baseline population; the population included in the analyses after 3 years of follow-up; and the lung cancer patients who were excluded during follow-up due to death or emigration. From now on, they will be referred to as the baseline, follow-up, and excluded populations, respectively. Additionally, we will present the number, percentage, and characteristics of lung cancer patients working and receiving disability pension after 3 years. Logistic regression was performed to compare the odds of working and receiving disability pension in the four SEP subgroups. Both crude and adjusted models for age, gender, and comorbidities were conducted, and the estimates are presented as odds ratios (ORs) with 95% confidence intervals (CIs). Finally, in the logistic regression, a test for trend was performed for household income, educational level, and prior sick leave by considering the subgroups as continuous variables. STATA version 17.0 was used as statistical software [33].

## Results

### Study population

**Figure 1:**
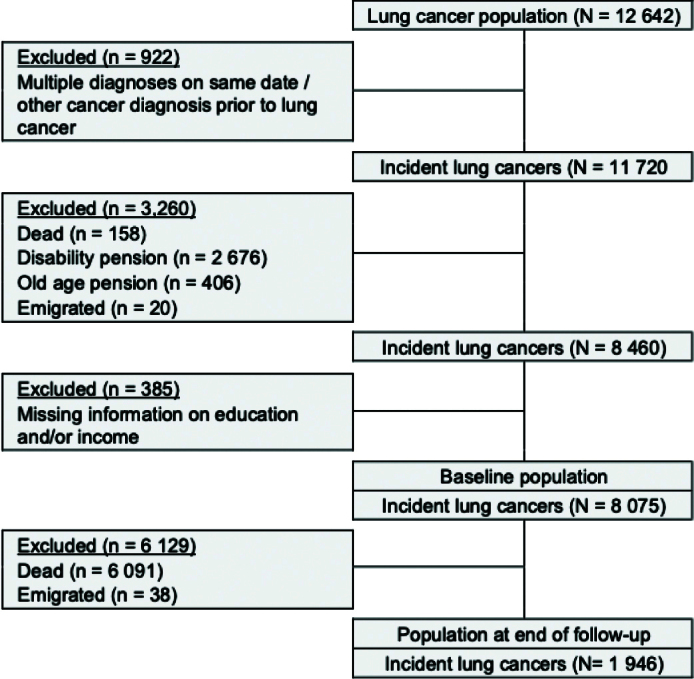
Flow chart of the exclusion process from initial to final study population.

A total of 12,642 Danish residents between the ages of 20 and 60 years with a lung cancer diagnosis were identified in CAR between January 2000 and December 2015. Of these, 8,075 lung cancer patients were eligible for inclusion. Exclusion criteria were: (1) multiple cancer diagnoses on the same date or having been diagnosed with another cancer before the lung cancer diagnosis (*n* = 922), (2) receiving age-related or disability pension at baseline (*n* = 3,260), and (3) missing information on income and/or education (*n* = 385). Moreover, 6,129 lung cancer patients who died or emigrated during follow-up were excluded, resulting in 1,946 patients available for analysis (Figure 1).

### Baseline characteristics

In the baseline population, most lung cancer patients were between 50 and 60 years of age, had no comorbidities and no previous sick leave, and were working 2 years before diagnosis (Table 1). The gender distribution was approximately equal, and most patients with lung cancer had a low or medium income and attained primary/high school or vocational education as their highest level of education.

**Table 1:** Baseline characteristics of study population at the time of inclusion. Presented for the baseline, follow-up, and excluded populations.

	Baseline population *n* (%)	Follow-up population *n* (%)	Excluded population *n* (%)
All	8,075 (100)	1,946 (100)	6,129 (100)
Age			
20–39	245 (3.0)	94 (4.8)	151 (2.5)
40–49	1,465 (18.1)	355 (18.2)	1,110 (18.1)
50–60	6,365 (78.8)	1,497 (76.9)	4,868 (79.4)
Gender			
Female	3,818 (47.3)	1,030 (52.9)	2,788 (45.5)
Male	4,257 (52.7)	916 (47.1)	3,341 (54.5)
Comorbidity 5 years before			
No	7,096 (87.9)	1,689 (86.8)	5,407 (88.2)
Yes	979 (12.1)	257 (13.2)	722 (11.8)
Household income			
Low	3,317 (41.1)	656 (33.7)	2,661 (43.4)
Medium	4,052 (50.2)	1,068 (54.9)	2,984 (48.7)
High	706 (8.7)	222 (11.4)	484 (7.9)
Highest attained education			
Primary & high school	3,176 (39.3)	771 (39.6)	2,405 (39.2)
Vocational education	3,496 (43.3)	798 (41.0)	2,698 (44.0)
Short further education	243 (3.0)	59 (3.0)	184 (3.0)
Bachelor’s degree	902 (11.2)	241 (12.4)	661 (10.8)
Long further education	258 (3.2)	77 (4.0)	181 (3.0)
Sick leave 2 years before diagnosis (weeks)			
0	6,497 (80.5)	1,561 (80.2)	4,936 (80.5)
1–7	858 (10.6)	212 (10.9)	646 (10.5)
8–27	492 (6.1)	120 (6.2)	372 (6.1)
≥28	228 (2.8)	53 (2.7)	175 (2.9)
Work status 2 years before diagnosis			
Working	6,915 (85.6)	1,712 (88.0)	5,203 (84.9)
Not working	1,160 (14.4)	234 (12.0)	926 (15.1)

The lung cancer patients in the follow-up population had higher income, were more often women, and worked 2 years before diagnosis compared with the population of excluded lung cancer patients (Table 1).

### Work status

Of the 1,946 patients with lung cancer available for analyses, 45.9% were not working 3 years after diagnosis (Table 2). These patients were more often women, older, and had both lower income and educational levels compared with the lung cancer patients who were working. Moreover, among the lung cancer patients who were not working, a more significant percentage had sick leave before diagnosis and were not working 2 years before diagnosis.

**Table 2:** Associations between socioeconomic factors at baseline and working 3 years after diagnosis.

	Working	Not working	Crude	Adjusted model**	Test for trend
	*n* (%)	*n* (%)	OR (95%-CI)	OR (95%-CI)	*P*
All*	1,052 (54.1)	894 (45.9)			
Age					
20–39	56 (5.3)	38 (4.3)			
40–49	215 (20.4)	140 (15.7)			
50–60	781 (74.2)	716 (80.1)			
Gender					
Female	526 (50.0)	504 (56.4)			
Male	526 (50.0)	390 (43.6)			
Comorbidity 5 years before					
No	910 (86.5)	779 (87.1)			
Yes	142 (13.5	115 (12.9)			
Household income					<0.001
Low	353 (33.6)	303 (33.9)	Ref.	Ref.	
Medium	537 (51.1)	531 (59.4)	0.87 (0.71, 1.05)	0.88 (0.72, 1.07)	
High	162 (15.4)	60 (6.7)	2.32 (1.66, 3.24)	2.31 (1.65, 3.24)	
Highest attained education					<0.01
Primary & high school	413 (39.3)	358 (40.0)	Ref.	Ref.	
Vocational education	410 (39.0)	388 (43.4)	0.92 (0.75, 1.12)	0.89 (0.73, 1.08)	
Short further education	34 (3.2)	25 (2.8)	1.18 (0.69, 2.01)	1.07 (0.62, 1.83)	
Bachelor’s degree	141 (13.4)	100 (11.2)	1.22 (0.91, 1.64)	1.26 (0.93, 1.70)	
Long further education	54 (5.1)	23 (2.6)	2.03 (1.22, 3.38)	1.92 (1.15, 3.21)	
Sick leave 2 years before index date (weeks)					<0.001
0	892 (84.8)	669 (74.8)	Ref.	Ref.	
1–7	98 (9.3)	114 (12.8)	0.64 (0.48, 0.86)	0.64 (0.48, 0.86)	
8–27	49 (4.7)	71 (7.9)	0.52 (0.35, 0.76)	0.51 (0.35, 0.75)	
≥28	13 (1.2)	40 (4.5)	0.24 (0.13, 0.46)	0.25 (0.13, 0.46)	
Working status 2 years before index date					
Working	979 (93.1)	733 (82.0)	Ref.	Ref.	
Not working	73 (6.9)	161 (18.0)	0.34 (0.25, 0.45)	0.32 (0.24, 0.43)	

OR: odds ratio; CI: confidence interval.

* % of total population after end of follow-up.

** Adjusted for age, gender, and comorbidities.

The adjusted analyses showed that lung cancer patients with high income had significantly higher odds of working 3 years after diagnosis compared to lung cancer patients with low income (Table 2). For educational level, only lung cancer patients with long further education had higher odds of working 3 years after diagnosis compared with lung cancer patients with primary/high school. However, there was a trend toward increasing odds of working with a higher level of education (*p* < 0.001). The odds of working decreased with more extended periods of sick leave before diagnosis. This showed a statistically significant negative trend (*p* < 0.001). Moreover, not working 2 years before diagnosis was associated with significantly lower odds of working after 3 years. Similar estimates were found in both the crude and adjusted models.

### Disability pension

Of the 1,946 lung cancer patients in the follow-up population, 407 (20.9%) received a disability pension within 3 years after diagnosis (Table 3). Those who received disability pension were older and had lower income and lower educational levels than the lung cancer patients who did not receive disability pension. They also had more sick leave before diagnosis, and fewer patients worked 2 years before diagnosis compared to patients who did not receive a disability pension.

**Table 3:** Associations between socioeconomic factors at baseline and disability pension 3 years after diagnosis.

	Disability pension	No disability pension	Crude	Adjusted model**	Test for trend
	*n* (%)	*n* (%)	OR (95%-CI)	OR (95%-CI)	*P*
All*	407 (20.9)	1,539 (79.1)			
Age					
20–39	14 (3.4)	80 (5.2)			
40–49	62 (15.3)	293 (19.0)			
50–60	331 (81.3)	1,166 (75.8)			
Gender					
Female	223 (54.8)	807 (52.4)			
Male	184 (45.2)	732 (47.6)			
Comorbidity 5 years before					
No	345 (84.8)	1,344 (87.3)			
Yes	62 (15.2)	195 (12.7)			
Household income					0.01
Low	179 (44.0)	477 (31.0)	Ref.	Ref.	
Medium	213 (52.3)	855 (55.6)	0.66 (0.53, 0.83)	0.67 (0.53, 0.84)	
High	15 (3.7)	207 (13.5)	0.19 (0.11, 0.34)	0.19 (0.11, 0.33)	
Highest attained education					<0.01
Primary & high school	178 (43.7)	593 (38.5)	Ref.	Ref.	
Vocational education	174 (42.8)	624 (40.6)	0.93 (0.73, 1.18)	0.95 (0.75, 1.20)	
Short further education	12 (3.0)	47 (3.1)	0.85 (0.44, 1.64)	0.89 (0.46, 1.72)	
Bachelor’s degree	37 (9.1)	204 (13.3)	0.60 (0.41, 0.89)	0.61 (0.41, 0.90)	
Long further education	6 (1.5)	71 (4.6)	0.28 (0.12, 0.66)	0.30 (0.13, 0.70)	
Sick leave 2 years before diagnosis (weeks)					<0.001
0	296 (72.7)	1,265 (82.2)	Ref.	Ref.	
1–7	52 (12.8)	160 (10.4)	1.39 (0.99, 1.95)	1.40 (1.00, 1.96)	
8–27	34 (8.4)	86 (5.6)	1.69 (1.11, 2.56)	1.43 (1.09, 2.53)	
≥28	25 (6.1)	28 (1.8)	3.82 (2.19, 6.64)	3.73 (2.14, 6.50)	
Work status 2 years before diagnosis					
Working	317 (77.9)	1,395 (90.6)	Ref.	Ref.	
Not working	90 (22.1)	144 (9.4)	2.75 (2.06, 3.68)	2.88 (2.14, 3.87)	

OR: odds ratio; CI: confidence interval.

* % of total population after end of follow-up.

** Adjusted for age, gender, and comorbidities.

The adjusted analyses showed that the odds of receiving disability pension decreased with higher income (*p* = 0.01), as both medium and high income had significantly lower odds of disability pension compared with lung cancer patients with low income. A similar trend was found regarding educational level (*p* < 0.01). However, the estimates were only statistically significant for lung cancer patients with a bachelor’s degree and long further education compared to primary/high school.

For prior employment patterns, the odds of disability pension increased with more extended periods of sick leave (*p* < 0.001), and being out of work 2 years before diagnosis was associated with significantly higher odds of disability pension when compared with the lung cancer patients who were working 2 years before diagnosis. The associations in the crude analysis remained unchanged when adjusted for age, gender, and comorbidities.

## Discussion and conclusion

This historical register-based cohort study is the first to investigate the association of four important variables of SEP on work status and disability pension in patients with lung cancer. Household income, educational level, and prior employment patterns, including sick leave and work status, were associated with work status and disability pension 3 years after diagnosis. Socioeconomically disadvantaged lung cancer patients had lower odds of working and higher odds of receiving disability pension than their better-off counterparts.

Previous cohort studies have shown that employment status and sick leave prior to diagnosis can impact post-cancer employment [29]. A Norwegian cohort study of 2008 cancer patients (24 lung cancer patients) found that >30 days of sick leave the year before diagnosis was associated with a higher rate of sick leave 5 years after diagnosis [34]. A similar association was demonstrated for employment status 5 years after cancer [7]. However, none of these studies distinguished between diagnoses. In this study, we also found that employment patterns, that is, not working and having sick leave before diagnosis, were associated with significantly lower chances of working and a higher risk of receiving disability pension 3 years after lung cancer. Moreover, previous research on cancer in general has found income to impact employment status after cancer [30]. Still, few studies have investigated the association between income and employment status in patients with lung cancer. A German cross-sectional study found a trend of increasing return to work rates for those with higher household incomes [21]. This is consistent with our findings, which showed higher odds of working among lung cancer patients with high income compared with those with low income. Finally, the present study found increasing odds of working and decreasing odds of disability pension among those with a higher level of education. Several studies have shown a similar impact of educational level on the employment of cancer patients overall [6, 30].

A recent review examining social vulnerability indices and their ability to predict poor health outcomes identified income, education, and social support as the primary domains contributing to social vulnerability, emphasizing the significance of these variables [35]. In the present study, four distinct indicators were used to assess SEP, including income and education. Although social support is recognized as a highly relevant factor with regard to health outcomes, the register-based design of this study limited the ability to include variables representing this domain. Nevertheless, education, income, and employment patterns are considered to represent essential aspects of the multidimensional construct of SEP [36]. Each variable describes both distinct and overlapping mechanisms through which SEP may affect health outcomes and the ability to work [10]. Many patients with lung cancer experience symptoms like dyspnea, pain, and fatigue up to 5 years after diagnosis [37, 38]. Symptoms have all been proven to affect the ability to work in cancer patients [39–41]. Evidence shows that rehabilitation can reduce the symptom burden. However, it could be hypothesized that individuals with limited economic latitude are less likely to seek professional support and, thus, may face more difficulties with employment after cancer. This may also be explained by their low health literacy [42, 43], which can negatively affect their ability to navigate their health situation and communicate with healthcare professionals [44]. As a result, lung cancer patients with low health literacy could be less likely to express needs concerning their health and work situation and, thus, not be referred to rehabilitation services. This hypothesis is supported in existing research, showing that cancer patients with low SEP more often report unmet rehabilitation needs [45] and are less often referred to and receive rehabilitation services [14]. Another important point to discuss is that the reduced physical capacity experienced by lung cancer patients can have a more negative impact on those with physically demanding jobs [46–48]. It is likely that lung cancer patients with less education are more often employed in physically demanding jobs, which could partially explain the gradient of worse work outcomes found in the present study. On the other hand, common impairments in lung cancer patients, such as fatigue and depression [49], can affect work performance in many types of jobs, including non-manual jobs [39]. However, adjusting work tasks and providing flexibility, which can support the return to work [6], are likely to be more attainable in jobs of higher occupational classes [8].

Collectively, our findings showed that socioeconomically disadvantaged lung cancer patients have an increased risk of adverse employment outcomes, underlining the need for more attention on this specific group of patients. More research on the mechanisms behind the observed disparities is needed to develop strategies to reduce the social gradient in employment after lung cancer. The presence of a similar social gradient in the incidence and management of lung cancer suggests a deeper underlying issue [13, 50]. This calls for increased attention to initiatives that address prevention, structural factors, and rehabilitation with a focus on social differentiation.

### Strengths and limitations

The use of the well-documented Danish registers ensured high-quality data. It allowed for complete follow-up of all relevant study participants, which, along with the longitudinal design of the study, resulted in a low risk of information and selection bias. Moreover, we were able to consider several essential covariates as confounding factors. However, this study has some limitations that must be considered. First, we lacked information on several potential confounders, including psychological comorbidities, marital status, occupation, social support, and cancer-related factors such as stage of disease and received treatment [29, 30, 46]. The inability to adjust for these factors could have confounded the results. Second, the use of disposable household income as the classification of SEP poses a risk of misclassification. Financial losses, inherent in, for example, company accounts or sales of stocks, affect the total disposable income negatively and can even lead to negative disposable household income in some instances. Individuals registered with a negative income were classified as having a low income, that is, low SEP, despite possibly having a ‘true’ high SEP. This misclassification may introduce information bias, thereby obscuring the association. Third, for assessing comorbidity status, we included codes from DNPR, which requires hospitalization, thus reflects the most severe cases. We therefore did not have information on patients with mild disease, who are only treated in primary care, potentially leading to an underestimation of the number of patients with comorbidities and leading to residual confounding.

### Conclusion

In conclusion, our study provides updated evidence of the association between SEP and employment status in lung cancer patients. We found that socioeconomically disadvantaged lung cancer patients who defined in terms of low income, short educational level, and previous history of sick leave and unemployment before cancer diagnosis have lower odds of working and have higher odds of receiving disability pension 3 years after diagnosis compared to their better-off counterparts. Thus, this study highlights the need for increased attention on socioeconomically disadvantaged lung cancer patients to ensure that adequate information and the correct interventions are provided to support them in returning to work.

## Data Availability

Data are not available due to legal restrictions as data are stored on a server at Statistics Denmark.
